# The Italian Family Satisfaction in the Intensive Care Unit Questionnaire: A Psychometric Evaluation Using the Rasch Model

**DOI:** 10.3390/healthcare11141997

**Published:** 2023-07-11

**Authors:** Matteo Danielis, Renzo Zanotti, Marika Rosset, Serena Giorgino, Sara Gentilini, Dina Molaro, Anna Qualizza, Alessandro Garau

**Affiliations:** 1 Laboratory of Studies and Evidence Based Nursing, Department of Medicine, University of Padua, 35131 Padua, Italy; renzo.zanotti@unipd.it; 2 Department of Anaesthesia and Intensive Care, Academic Hospital of Udine, 33100 Udine, Italy; marika.rosset@asufc.sanita.fvg.it (M.R.); serena.giorgino@asufc.sanita.fvg.it (S.G.); sara.gentilini@asufc.sanita.fvg.it (S.G.); dina.molaro@asufc.sanita.fvg.it (D.M.); anna01.qualizza@asufc.sanita.fvg.it (A.Q.); alessandro.garau@asufc.sanita.fvg.it (A.G.)

**Keywords:** intensive care unit, family satisfaction, quality improvement, psychometrics, Rasch analysis

## Abstract

Quality measurement of the intensive care unit (ICU) should include families’ perspectives, their satisfaction with the care process and outcomes, and the evaluation of actions to improve their psychological health and wellbeing. The current study was designed to validate the Italian version of the Family Satisfaction in the Intensive Care Unit (FS-ICU) using the Rasch model. Results included reliability and separation for items and persons, item fit statistics, unidimensionality, and item characteristic curve. The study was conducted between August 2022 and February 2023. A total of 108 family members (mean age 54.9 years) completed the FS-ICU questionnaire. The instrument had a moderate discrimination ability and only five items (#21, #23, #10, #22, and #24) exhibited a misfit. The Rasch dimension explained 52.1% of the variance in the data, while the unexplained variance in the first contrast is 7.2%, which indicates a possible second dimension. FS-ICU was shown to be beneficial as an assessment instrument for family member satisfaction in the ICU, despite some flaws that need to further be addressed to improve the scale.

## 1. Introduction

One of the nursing-sensitive outcomes that has received less attention in the literature is the experience of being in an intensive care unit (ICU), particularly from the perspective of family members [1]. After the extraordinary measures imposed during the COVID-19 pandemic [2], when the ICU restored its previous practice, that is when visitation restrictions were standard procedures, increasing visitor accessibility and involving family members in patient care during hospitalization were thought to be essential to raising awareness of and readiness for the care needed after ICU discharge [3,4]. In response to this situation, hospitals have developed several techniques to balance patient safety concerns and family needs. Furthermore, family satisfaction (FS) with the ICU is acknowledged as a sign of the quality of treatment, and family member points of view help healthcare professionals (HCPs) adapt the care provided [5].

Self-reported surveys (such as the Critical Care Family Satisfaction Survey (CCFSS) [6]), and those based on structured interviews (such as the Cuthbertson bereavement follow-up service [7]) are two sets of tools used for the evaluation of FS in the ICU. Four surveys—the Critical Care Family Needs Inventory (CCFNI), the Society of Critical Care Medicine Family Needs Assessment (SCCMFNA), the CCFSS, and the Family Satisfaction in the Intensive Care Unit (FS-ICU)—were rated as level I (well-established quality) [5]. The CCFNI and FS-ICU are the most psychometrically robust of these four instruments. The FS-ICU assesses satisfaction with care and decision-making, whereas the CCFNI measures needs met.

The FS-ICU, created by Heyland and Tranmer in 2001 [8] and further refined by Wall et al. in 2007 [9], has been released in a variety of languages and versions (e.g., Norway [10]). To date, the Italian version of the questionnaire has been validated by face and content validation methods [11]. The internal consistency assessed by Cronbach’s alpha ranged between 0.944 and 0.962. However, to our knowledge, no fully validated FS-ICU tool is available.

Despite the lack of a standardized definition, FS in the ICU is generally defined as overall family satisfaction in terms of matching family members’ expectations and needs in the ICU. Moreover, a number of factors including communication, atmosphere, visitation policies, decision-making, and emotional support are linked to family satisfaction in the ICU [12]. A thorough understanding of FS response rates may help HCPs and other stakeholders to improve the experience of families of patients in the ICU and to support family members who may have a negative ICU experience, and could ultimately reflect on low satisfaction levels.

This study provides further psychometric data on the FS-ICU instrument by applying Rasch analysis, and to measure how families evaluate their satisfaction with the ICU stay in an Italian context during the post-pandemic period. Compared to other items response theory (IRT) models, the Rasch analysis model assumes that the probability of a correct response depends on the person’s ability and difficulty [13]. This feature contributes to the uniqueness and utility of the Rasch analysis model in providing objective measurement, facilitating meaningful comparison, and identifying bias of existing instruments. Rasch analysis considers a questionnaire as a whole, offering graphic examples of how certain items might be reweighed or eliminated to better suit the data and also to focus on the specific requirements that the questionnaire intends to measure. Although Rasch analyses were primarily developed and used for educational assessment, they are now increasingly being used in health research. To our knowledge, no Rasch analysis of the FS-ICU has yet been performed.

## 2. Materials and Methods

### 2.1. Design, Setting, and Sample Size

A cross-sectional design was adopted. Results are reported according to the Strengthening the Reporting of Observational Studies in Epidemiology (STROBE) checklist for cross-sectional studies [14].

An Academic Hospital in an easternmost region of Italy was approached and the study was conducted between August 2022 and February 2023. The ICU setting was a conventional open space area with some separate rooms for special conditions (infective patients), equipped with 16 beds. Registered nurses (RNs), physicians, and nursing assistants (NAs) made up the personnel. Typically, the ICU has a critical care physician to patient ratio of 1:6 and a RN to patient ratio of 1:2 per shift. Generally, the average ICU length of stay (LOS) is 5.0 days, and the average ICU occupancy rate is 80%. Before the COVID-19 outbreak, these ICUs adopted a flexible visiting policy (more than one hour a day, more than one visitor at a time), which was restricted during the pandemic waves (one hour a day, only one visitor at a time). In the study period, at the end of the COVID-19 pandemic, an ‘open’ visitation policy was reintroduced.

Rasch analysis does not require large sample sizes to produce reliable estimates [13], and previous studies have recommended a sample size of 100 respondents [15,16]. In determining the best sample size, we considered the characteristics of the construct being assessed (family satisfaction with ICU), that was not expected to have substantial variation; and some barriers to effective recruitment, e.g., limited resources (families asked for clarification and needed further explanation) and ethical issues (considering the timing of the approach at a challenging and stressful moment for families).

The inclusion criteria of the study were (a) being a family member and/or a person with whom the patient spends most of the time, (b) being a family member of a patient who was expected to be cared for at least three days in ICU to ensure adequate exposure to the ICU environment, (c) being willing to visit the patient daily, (d) being adult (18 years of age and older), and (e) being willing to participate in the study.

### 2.2. Data Collection

Family members meeting the inclusion criteria were approached by a HCP who was working in the ICU and was a member of the research team. Following an explanation of the study and an oral agreement to participate, participants received an Italian-translated version of the FS-ICU questionnaire together with a cover letter outlining the survey’s goals [11]. Patients’ demographics (age, gender), clinical parameters (diagnosis, comorbidities, Glasgow Coma Scale (GCS) and Richmond Agitation-Sedation Scale (RASS) scores), anthropometric measures (weight and height, both required to calculate the body mass index (BMI)), and previous habits (smoking) were assessed. Altogether, information regarding the family member—age, gender, education, relation to patient, prior experience in ICU, and living situation—was collected in an ad hoc form. Then, within 48 h of a patient’s ICU discharge, data were collected to evaluate families’ satisfaction.

### 2.3. Instrument

Family members’ satisfaction was investigated with the Italian FS-ICU questionnaire [8,11], which comprises of two sections regarding the satisfaction with the care received (16 items assessing patient care, family care, caregivers’ professional competence, and ICU environment) and the decision-making process (10 items assessing information needs and family needs, involvement in decision-making). All items are scored on a five-point Likert scale (from 1 = very dissatisfied, to 5 = very satisfied). In line with previous research [10,17], the total scores were then transformed into a 0–100 scale, where 0 is least satisfied and 100 is most satisfied. Data were collected from all participants via self-administered questionnaires in written form. The validated Italian version of FS-ICU was already available for use (https://fsicu.org/professionals/survey/versions/ (accessed on 28 July 2022)).

### 2.4. Validation Process of the Instrument

Reliability and separation for items and persons, item fit statistics, unidimensionality and item characteristic curve were assessed using the Rasch model [13].

The indices for person separation index, person reliability, item separation, and item reliability were used to assess reliability. The separation index reflects the number of unique strata into which the sample or items can be subdivided and provides an assessment of the distribution of items or people along the construct continuum. Typically, the minimum requirement for the index is ≥2, which shows that the instrument can separate persons from at least two strata, for example, low and high ability [13]. A person reliability of 0.8 represents a good level of separation and is considered the minimum preferable value [15].

Infit and outfit statistics were used to analyze individual item and person fit to see how well the data matched the Rasch model. A value between 0.5 and 1.3 for infit and outfit statistics would be productive and useful [18]. Mean square (MNSQ) and Z-standardized scores (ZSTD) for each of these fit statistics were calculated [15]. Wright Maps were used to visualize the relationship between items and persons [19]. Every item was also examined for Differential Item Functioning (DIF) between females and males. A noticeable DIF was defined according to two criteria: (1) DIF contrast > 0.5 logits and (2) significance of the difference (*p* < 0.05) [15].

Results were deemed to suggest unidimensionality if the Rasch dimension explained >60% of the variance and the unexpected variance that accounted for the first contrast was 5% [15]. Less than 2 was deemed the optimal eigenvalue of the first contrast in the principal component analysis on standardized residuals to establish unidimensionality, while less than 3 was deemed tolerable [20]. The element that explains the most variance in the residuals is called the “first contrast”.

As presented here, the item characteristic curve (ICC), which is defined as an ogive-shaped plot of the probabilities of a correct response to an item for any value of the underlying trait in a respondent, is used to describe the relationship between the test response probability and the level of the intrinsic trait [21].

### 2.5. Analysis

Data were entered into a Microsoft Excel^®^ worksheet. While continuous variables (e.g., age, length of stay) were displayed as mean, standard deviations (SD), and median, nominal variables (e.g., gender, reason for admission) were shown as absolute frequencies and percentages. The Statistical Package for Social Sciences (SPSS) v.27 was used for data entry, dataset management, and descriptive statistics. This study used WINSTEPS to analyze the data for the validation process (Winsteps Rasch Measurement Analysis; Version 5.5.0.0, Chicago, IL, USA). All tests were 2-sided with an alpha level of 0.05.

### 2.6. Ethical Considerations

The Regional Ethics Committee of Friuli Venezia Giulia (Italy) approved the data collection (CEUR-2020-Sper-012). The study was conducted according to the criteria set by the Declaration of Helsinki. In addition, each family member/caregiver provided written informed consent after having received appropriate information regarding the research aims and procedures. The researchers ensured confidentiality during each data handling process. Moreover, participants were free to withdraw from the study at any time without providing reasons.

## 3. Results

### 3.1. FS-ICU Scores

From August 2022 to February 2023, family members of 119 ICU patients agreed to participate in this study; 108 questionnaires were returned. The mean score of the Acute Physiologic Assessment and Chronic Health Evaluation II (APACHE II) of the enrolled patients was 16.2 (8 to 23), with an ICU survival rate of 87.9% (Table 1). Patients were mostly men (63.9%), with a mean age of 66.3 years. Among family members, most respondents, whose mean age was 54.9 years, were the daughter/son of the patient (40.7%), and lived with the patients before the ICU (50.0%, Table 2). Satisfaction Score was 84.8 ± 11.6 (FS-ICU/Total). Sub scores satisfaction with overall care was 86.1 ± 12.1 (FS-ICU/Care) and satisfaction with decision-making process was 82.8 ± 12.4 (FS-ICU/DM). Families reported the greatest satisfaction with the ICU staff’s treatment of patient’s breathlessness (item #2b, Table 3). The item with the lowest scores was the ICU waiting room atmosphere (item #10).

### 3.2. Reliability

The Person Separation Index (PSI) is 2.95, indicating the ability of the dataset to distinguish between three distinct groups of people with different levels of latent trait based on their responses [15]. Generally, higher separation indices indicate better discrimination and measurement that is more reliable. The Person Reliability (PI) of 0.90 suggests that the items consistently measured the latent trait and provided reliable information about individuals’ positions on those traits. The item separation index is 4.53, and the item reliability is 0.95. An interesting aspect is that the confidence about the measures of items is much higher than that for persons. This indicates that there are more than four levels of test items in terms of difficulty. These values suggest that the sample was large enough to confirm the item difficulty hierarchy (i.e., construct validity).

### 3.3. Item Fit and Gender Invariance

The items were ordered based on their fit statistics. Infit is sensitive to inliers, outfit is sensitive to outliers. The range of values in our data was 0.54–2.22. These statistics indicate the extent to which individuals’ items fit with the overall pattern of responses or whether they deviate from the expected response patterns. Table 4 below presents items #21, #23, #10, #22, and #24 with higher infit and outfit values compared to the other items. These items also had the lowest scores in terms of satisfaction, which is indicative of the experiences of the family members. In addition, no significant DIF was found for female and male by gender, except for item q23.

### 3.4. Unidimensionality

**Figure 1:**
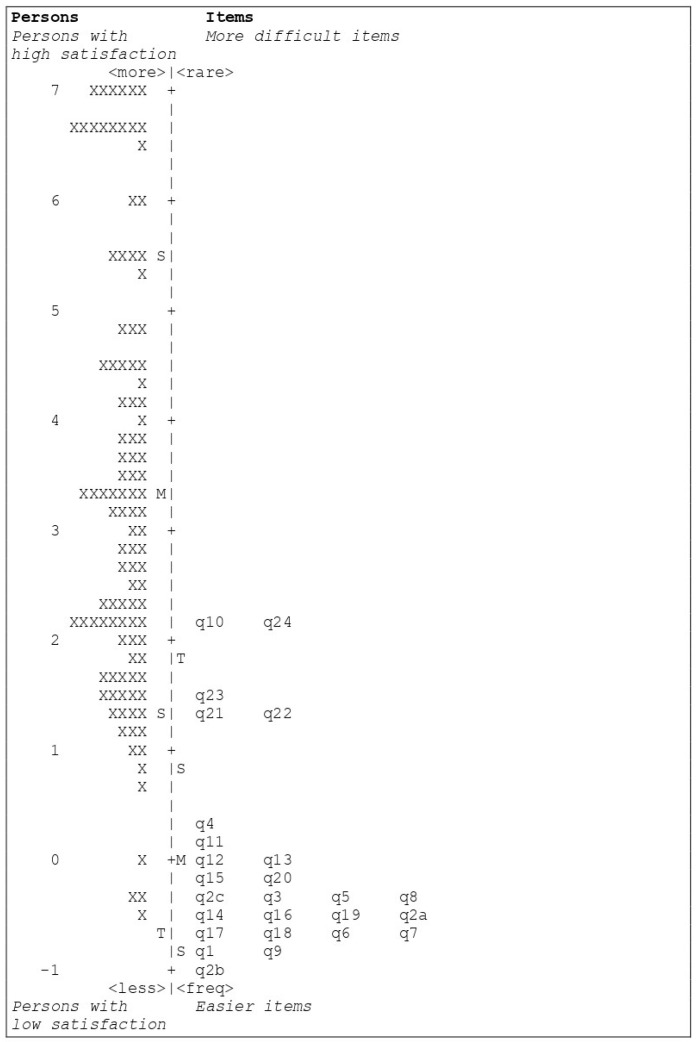
Item map (FS-ICU). M = Mean persons’ ability or mean items’ difficulty; S = one standard deviation; T = two standard deviations. Each ‘X’ represents one participant. The vertical line is a continuum representing the measures of persons’ ability (left side) and items’ difficulty (right side), plotted in logit units. The persons’ ability and items’ difficulty increase from the bottom to the top.

Figure 1, which presents the item map, evaluates the unidimensionality of the measurement instrument. The item map displays the positioning of each item based on its measure and on the participant’s responses. The Rasch dimension explained 52.1% of the variance in the data (Eigenvalue = 28.3). The raw variance explained by items is only 19%, while the unexplained variance in the first contrast is rather sizeable (7.2%, Eigenvalue = 3.89), which indicates a possible second dimension.

### 3.5. Item Characteristics Curves

**Figure 2:**
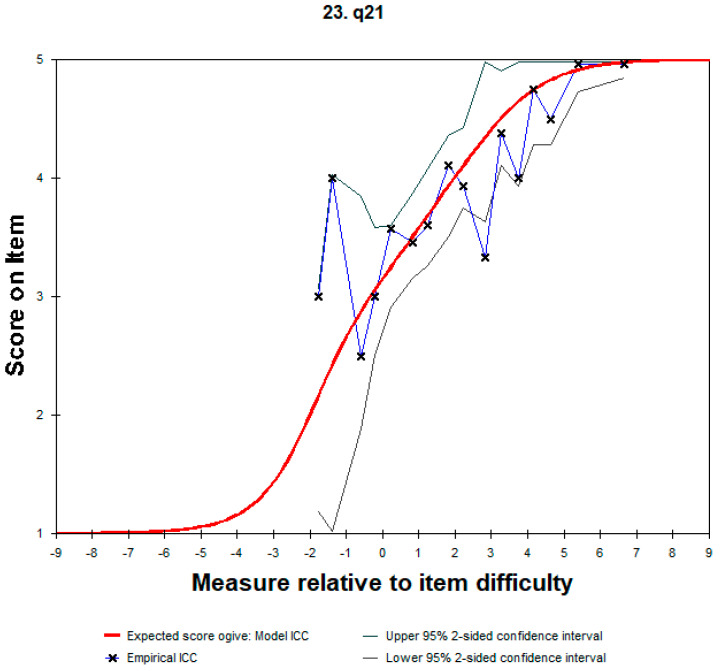
Item 21 characteristic curve.

Based on Supplementary Figure S1, the empirical ICC curves fit well with the expected ICC curves for most of the items of the FS-ICU. The red line is the item characteristic curve as expected by the Rasch model and the blue line is the empirical ICC, while “X” are the means of the measures and ratings for observations in the interval. When the “X” on the blue line is at, or very close to, the red line, the test is a good fit to the model. The green-gray lines are two-sided, 95% confidence bands. Figure 2 shows item #21’s curve, which fails to fit as expected and exceed the 95% confidence interval.

## 4. Discussion

This study investigated the psychometric properties of the FS-ICU instrument using Rasch analysis to examine whether the FS-ICU may be a suitable tool to collect data on satisfaction among families from an Italian ICU. Participants were adult family members/caregivers who visited their loved ones daily. According to the results, family members were usually satisfied with the ICU. Family members rated both nursing care and the entire treatment (FS-Care) together with the decision-making process (FS-DM) as satisfactory, in agreement with most of the recent research on this topic.

The PSI of 2.95 suggests that the items in the questionnaire are moderately effective in discriminating individuals with different levels of latent trait being measured (i.e., how well the ICU staff treated family members). Higher PSI indicated better discrimination [22]. Additionally, the person reliability of 0.90 showed that the measurement of person on the latent trait has good internal consistency and provides reliable information on individuals’ positions on that trait. This means that the instrument is consistent in measuring the construct of interest.

Item misfit can arise due to different measurement problems, including poor item quality. Item #10 is about the atmosphere of the ICU waiting room. As documented in the related literature, waiting spaces play an important role in the patient and family experience [23], so we recommend not removing this item. In our context, the waiting room is far from the ICU and is not well signposted. Those who do not use it have to wait in line in front of the ICU door. This may have confused the respondents, and each of them interpreted ‘waiting room’ in different ways.

Items #21, #22, #23, and #24 all relate to the decision-making process. Families were happier when they received clear and honest information in understandable language, which then allowed them to actively participate in decision-making [24,25]. A recent Chinese study on 548 family members in ICU—aimed at comparing the family–clinician shared decision-making interventions with standard care—showed improved families’ satisfaction (*p* = 0.0001) and reduced depression (*p* = 0.005), shortened patient stay in the ICU (*p* = 0.0004), and improved collaboration between HCPs (*p* < 0.05) [26]. It should be made understandable to both HCPs and family members what ‘decision-making’ involves. In other words, what the family member expects to make decisions on and what the HCPs want the family member to decide should be explained. This may have caused confusion in the aforementioned items and family members responded based on their own particular expectations, readiness, and preparedness for involvement [27]. Additionally, the decision-making concepts may have differed depending on the personal characteristics, such as age, education, and attitudes. We suggest rewording these elements by simplifying the term ‘decision-making’ and giving more examples to inform decisions based on the context of individual ICU teams, the needs of family members, available resources, and organizational features.

The raw variance explained by items was 19.0% and the explained variance in the first contrast was 7.2%. While the Rasch dimension dominates (almost three times the secondary dimension), the secondary dimension is noticeable. Based on the criteria provided by Linacre [15], the eigenvalue of 3.89 would suggest that there are about four items that constitute a second dimension.

Actually, the questionnaire was originally designed with two conceptual sections: the first part focuses on satisfaction with care and the second part assesses satisfaction with decision-making [8]. The study by Wall et al. (2007) performed a factor analysis using a two-factor model and found that four items that assessed information exchange loaded equally on both factors [9]. This suggested that the degree to which family members’ information needs are met in the ICU could also affect family members’ satisfaction with care. Another study aimed at exploring the psychometric properties of the FS-ICU in the Norwegian context [17] reported a different pattern (care and support for family members vs. care for patients) compared to the distribution of items on the two subscales in the original instrument. Given this, we considered the construct (family satisfaction with the ICU) as a whole, rather than treating the two sections (FS-Care and FS-DM) as separate tools. Our results suggest the possible emergence of a second dimension latent in the measure. It can be quite challenging to find a pattern among items that fit poorly in order to determine the type of a second dimension. These challenges should be given top priority in future study.

### 4.1. Implications and Future Research

Some implications and recommendations can be made from this research. First, despite having a moderate discrimination ability, there is room for improvement to enhance the instrument’s ability to distinguish more precisely between individuals with different levels of the latent trait. This can be achieved by including items with stronger discriminatory power or refining the wording of existing items. Secondly, the high person reliability indicates good internal consistency and reliability of the instrument. However, it is still important to monitor and evaluate the reliability over time to ensure consistent measurement quality. Then, given the presence of potential misfitting items, a detailed examination of these items is necessary. Investigating the reasons for the misfit, such as assessing the clarity of the item language or potential response biases, can determine whether or not to redistribute the items, or add or delete them. To improve the overall measurement quality, it is advisable to conduct further analysis, including item-level psychometric evaluations (e.g., item discrimination, item difficulty) and a thorough review of the measurement model used. Lastly, it is crucial to involve the HCPs and gather feedback from stakeholders to refine the instrument and enhance its validity and reliability.

### 4.2. Limitations

This study has some limitations. First, the study sample consisted of family members who visited critically ill patients in a single ICU, and it is thought that this may reduce representativeness and limit the generalizability of the results and the recommendations. Second, this is a preliminary analysis; thus, we recommend that the misfit items in the FS-ICU are modified and tested as a proposed new form in an independent sample. Before suggesting any changes to these items, further investigation should be carried out to clarify why the respondents answered differently to those situations and whether the single item has a significant impact on the overall score.

## 5. Conclusions

The main contributions of the present study are to demonstrate the usefulness of the FS-ICU in measuring the satisfaction of family members of critically ill patients and to provide useful information to further improve the tool. This study determined that the Italian version of the FS-ICU is a valid and reliable instrument when used to measure the principal construct. However, some item misalignments regarding the waiting room and the decision-making process should be considered for further analysis.

## Figures and Tables

**Table 1:** Characteristics of patients.

Variable	*n* = 108 (100%)
Age (years), mean (SD)	66.3 (14.1)
Gender, *n* (%)	
Male	69 (63.9)
Female	39 (36.1)
BMI (kg/m^2^), mean (SD)	24.8 (6.1)
Reason of admission in ICU, *n* (%)	
Organ failure	58 (53.7)
Trauma	25 (23.1)
Cerebrovascular disease	17 (15.7)
Post-operative	8 (7.4)
At least one comorbidity, *n* (%)	72 (66.7)
Smoking habit, *n* (%)	69 (67.6)
APACHE 2 score, mean (SD)	16.2 (9.3)
RASS score, mean (SD)	−2.09 (1.8)
Length of stay in ICU (days), mean (SD)	13.8 (8.6)
Survivors, *n* (%)	95 (87.9)

SD, Standard Deviation; BMI, body mass index; ICU, intensive care unit; APACHE, Acute Physiology and Chronic Health Evaluation; RASS, Richmond Agitation-Sedation Scale.

**Table 2:** Socio-demographic characteristics of family members.

Variable	*n* = 108 (100%)
Age (years), mean (SD)	54.9 (12.1)
Gender, *n* (%)	
Female	72 (66.7)
Male	36 (33.3)
Relationship to patient, *n* (%)	
Daughter/son	44 (40.7)
Wife/husband or significant other	39 (36.1)
Sister/brother	10 (9.3)
Mother/father	7 (6.5)
Other degree of relatedness	8 (7.4)
Education, *n* (%)	
Secondary school	52 (48.6)
Primary school	28 (26.2)
Degree or above	27 (25.2)
Prior experience with ICU, *n* (%)	35 (32.4)
Cohabitation with patient, *n* (%)	54 (50.0)
If no cohabitation, how frequently do you visit your loved one, *n* (%)	
More than weekly	27 (52.9)
Monthly	15 (29.4)
Weekly	9 (17.6)

SD, Standard Deviation; ICU, intensive care unit.

**Table 3:** Family satisfaction at item and dimension level (https://fsicu.org/professionals/survey/versions/ (accessed on 28 July 2022)).

FS-ICU 24, Item Number	Mean (SD)
FS-CARE ^a^	
1. How satisfied are you with the courtesy, respect, and compassion your family member (the patient) was given?	4.58 (0.69)
2a. How well the ICU staff assessed and treated your family member’s pain?	4.50 (0.64)
2b. How well the ICU staff assessed and treated your family member’s breathlessness?	4.60 (0.56)
2c. How well the ICU staff assessed and treated your family member’s agitation?	4.44 (0.66)
3. How satisfied are you with how well the ICU staff showed an interest in your needs?	4.47 (0.66)
4. How satisfied are you with how well the ICU staff provided emotional support to you?	4.24 (0.79)
5. How satisfied are you with the teamwork of all the ICU staff that took care of your family member?	4.45 (0.70)
6. How satisfied are you with the courtesy, respect, and compassion you were given?	4.52 (0.69)
7. How satisfied are you with how well the nurses cared for your family member?	4.55 (0.66)
8. How satisfied are you with how often nurses communicated to you about your family member’s condition?	4.45 (0.69)
9. How satisfied are you with how well doctors cared for your family member?	4.58 (0.60)
10. How satisfied are you with the atmosphere (mood) in the ICU waiting room?	3.58 (1.12)
11. How satisfied are you with the atmosphere (mood) of the ICU?	4.32 (0.72)
12. How satisfied are you with your participation in daily rounds?	4.33 (0.69)
13. How satisfied are you with your participation in the care of your critically ill family member?	4.37 (0.78)
14. How satisfied are you with the level or amount of health care your family member received in the ICU?	4.47 (0.68)
FS-DM ^a^	
15. How satisfied are you with how often doctors communicated to you about your family member’s condition?	4.41 (0.71)
16. How satisfied are you with the willingness of the ICU staff to answer your questions?	4.49 (0.59)
17. How satisfied are you with how well the ICU staff provided you with explanations that you understood?	4.55 (0.60)
18. How satisfied are you with the honesty of information provided to you about your family member’s condition?	4.53 (0.63)
19. How satisfied are you with how well the ICU staff informed you what was happening to your family member and why things were being done?	4.48 (0.72)
20. How satisfied are you with the consistency of information provided to you about your family member’s condition?	4.39 (0.81)
21. How satisfied are you with the inclusion in decision-making?	3.90 (1.04)
22. How satisfied are you with the support during decision-making?	3.92 (0.92)
23. How satisfied are you with the control over the care?	3.87 (1.00)
24. How satisfied are you with the time to address concerns and questions when making decisions?	3.66 (0.89)
FS-CARE subtotal ^b^	86.1 (12.1)
FS-DM subtotal ^b^	82.8 (12.4)
FS-TOTAL ^b^	84.8 (11.6)

FS-ICU, family satisfaction during the intensive care stay; FS-DM, decision-making; SD standard deviation. ^a^ Scoring system: from 1 = very dissatisfied, to 5 = very satisfied. ^b^ Scoring system: 0 is least satisfied and 100 is most satisfied.

**Table 4:** Item fit statistics and gender invariance.

				Infit		Outfit		Gender Invariance ^b^	
Item	Abbreviated Item	Measure ^a^	SE	MnSq	Zstd	MnSq	Zstd	DIF Contrast	*p*-Value
21	Feel included in the decision-making process	1.33	0.16	2.22	6.22	2.06	5.02	−0.71	0.452
23	Feel control over the care of the patient	1.49	0.16	1.85	4.62	1.73	3.79	−0.93	0.036
10	Atmosphere of the ICU waiting room	2.24	0.16	1.68	3.72	1.67	3.70	−0.43	0.177
22	Feel supported during the decision-making process	1.31	0.16	1.49	2.93	1.43	2.39	−0.26	0.389
24	Adequate time to address concerns and answer questions	2.14	0.16	1.22	1.35	1.31	1.85	−0.46	0.927
17	Staff provided understandable explanations	0.74	0.20	0.91	0.57	1.26	0.82	0.61	0.206
4	How well the staff provided emotional support toward family	0.41	0.17	1.01	0.08	1.18	0.88	0.40	0.175
1	Courtesy, respect, and compassion by staff toward patient	0.90	0.20	1.15	0.97	0.91	−0.15	0.64	0.125
20	Consistency of information about patient’s condition	0.12	0.18	1.15	0.99	0.93	−0.20	−0.04	0.744
15	Frequency of communication by doctors	0.22	0.18	0.97	0.17	0.84	−0.54	0.00	1.000
5	Coordination and teamwork by staff	0.33	0.19	0.93	0.41	0.78	−0.70	0.00	1.000
13	Satisfaction with involvement in the care	0.01	0.18	0.92	0.47	0.79	−0.83	0.43	0.042
19	Completeness of information about what was happening	0.48	0.19	0.92	0.51	0.73	−0.88	0.00	0.227
7	Skill and competence of nurses	0.74	0.20	0.89	0.69	0.88	−0.26	0.18	0.600
18	Honesty of information provided about patient’s condition	0.64	0.20	0.83	−1.14	0.62	−1.25	0.00	0.535
6	Courtesy, respect, and compassion by staff toward family	0.63	0.19	0.82	−1.17	0.64	−1.20	0.55	0.441
8	Communication by nurses	0.38	0.19	0.81	−1.32	0.63	−1.41	0.12	0.068
11	Atmosphere of the ICU	0.12	0.18	0.75	−1.78	0.65	−1.63	0.63	0.654
2a	Management of pain	0.53	0.19	0.71	−2.04	0.68	−1.04	−0.46	0.365
2b	Management of breathlessness	0.95	0.21	0.64	−2.49	0.68	−0.83	−0.70	0.641
14	Satisfaction with the level or amount of care the patient received	0.42	0.19	0.67	−2.34	0.57	−1.62	0.11	0.319
9	Skill and competence of doctors	0.82	0.20	0.65	−2.45	0.48	−1.70	0.19	0.694
3	How well staff considered family needs	0.38	0.19	0.62	−2.87	0.52	−1.95	0.68	0.398
16	Willingness of staff to answer questions	0.52	0.19	0.62	−2.80	0.62	−1.36	0.69	0.166
2c	Management of agitation	0.31	0.19	0.61	−2.80	0.54	−1.85	0.39	0.049
12	Satisfaction with involvement in daily medical visit	0.08	0.18	0.57	−3.32	0.54	−2.25	0.05	0.645

MnSq, mean square standardized residuals; SE, standard error; Zstd, standardized Z-values; DIF, differential item functioning. ^a^ The estimate of the item difficulty; values are reported in logits. ^b^ DIF contrast across gender = difficulty for females − difficulty for males. Mantel–Haenszel chi-square value tests.

## Data Availability

All data are available upon request.

## Supplementary Materials

The following supporting information can be downloaded at: https://www.mdpi.com/article/10.3390/healthcare11141997/s1, Figure S1: Item characteristic curves of FS-ICU questionnaire.
