# Supplementary material for: The Italian Family Satisfaction in the Intensive Care Unit Questionnaire: A Psychometric Evaluation Using the Rasch Model
Source: Healthcare (Basel). 2023 Jul 11;11(14):1997. doi: 10.3390/healthcare11141997 (PMC10379138; doi:10.3390/healthcare11141997)
Supplement: Supplementary file 1 [file healthcare-11-01997-s001.zip › healthcare-2460215-supplementary.pdf]

Supplementary Figure S1. Item characteristic curves of FS-ICU questionnaire

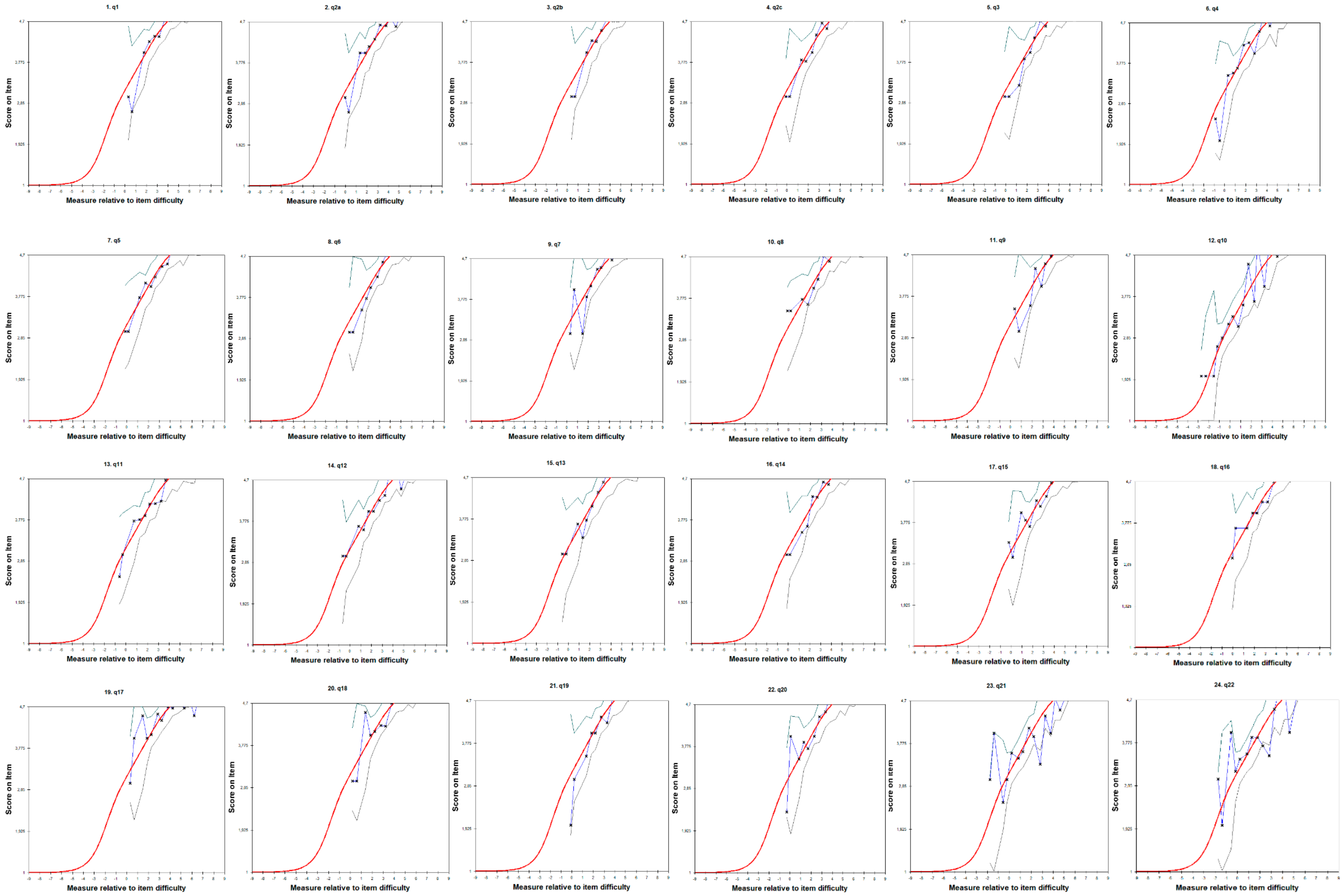

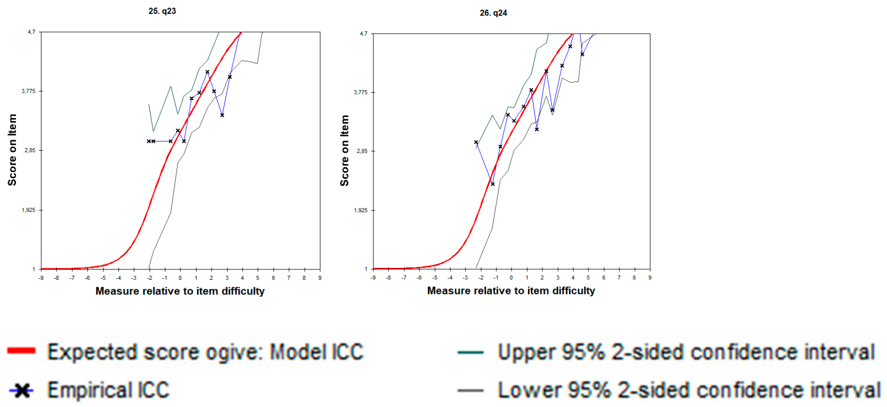
25. q23
26. q24
Score on Item
Measure relative to item difficulty
Expected score ogive: Model ICC
Empirical ICC
Upper 95% 2-sided confidence interval
Lower 95% 2-sided confidence interval
